# Intestinal epithelial suppressor of cytokine signaling 3 (SOCS3) impacts on mucosal homeostasis in a model of chronic inflammation

**DOI:** 10.1002/iid3.171

**Published:** 2017-05-15

**Authors:** Elisabeth J. Shaw, Emily E. Smith, Jayde Whittingham‐Dowd, Matthew D. Hodges, Kathryn J. Else, Rachael J. Rigby

**Affiliations:** ^1^ Division of Biomedical and Life Sciences Faculty of Health and Medicine Lancaster University Lancaster UK; ^2^ Faculty of Biology, Medicine, and Health Manchester University Manchester UK

**Keywords:** Epithelial cell, immunotherapy, intestine, mucosal homeostasis, SOCS3

## Abstract

**Introduction:**

Suppressor of cytokine signaling 3 (SOCS3) is a tumour suppressor, limiting intestinal epithelial cell (IEC) proliferation in acute inflammation, and tumour growth, but little is known regarding its role in mucosal homeostasis. Resistance to the intestinal helminth *Trichuris muris* relies on an “epithelial escalator” to expel the parasite. IEC turnover is restricted by parasite‐induced indoleamine 2,3‐dioxygenase (IDO).

**Methods:**

Mice with or without conditional knockout of SOCS3 were infected with *T. muris*. Crypt depth, worm burden, and proliferating cells and IDO were quantified. SOCS3 knockdown was also performed in human IEC cell lines.

**Results:**

Chronic *T. muris* infection increased expression of SOCS3 in wild‐type mice. Lack of IEC SOCS3 led to a modest increase in epithelial turnover. This translated to a lower worm burden, but not complete elimination of the parasite suggesting a compensatory mechanism, possibly IDO, as seen in SOCS3 knockdown.

**Conclusions:**

We report that SOCS3 impacts on IEC turnover following *T. muris* infection, potentially through enhancement of IDO. IDO may dampen the immune response which can drive IEC hyperproliferation in the absence of SOCS3, demonstrating the intricate interplay of immune signals regulating mucosal homeostasis, and suggesting a novel tumour suppressor role of SOCS3.

## Introduction

Intestinal epithelial cells (IEC) lining the gastrointestinal tract act as a first line of defence from the plethora of antigenic luminal content. This epithelium is in a constant state of renewal and repair which must be balanced to maintain barrier function whilst avoiding excessive growth. Disruption to this balance has been implicated in various diseases including inflammatory bowel disease (IBD) and bowel cancer 1.

Suppressor of cytokine signaling 3 (SOCS3) acts as a tumour suppressor and regulator of inflammatory signaling pathways. Decreased expression of SOCS3, often due to promoter hypermethylation, has been reported in a number of different cancers and cancer cell lines 2, 3, 4, 5. Colonic tumours are increased with regard to incidence and size in mice with IEC‐specific knockout of SOCS3 in an inflammation‐induced (azoxymethane/dextran sodium sulphate [DSS]) cancer model; while over‐expression of SOCS3 in vitro has been shown to result in decreased cell proliferation and wound repair 2, 6. SOCS3 is known to inhibit both IL‐6‐induced pSTAT3 and TNFα‐induced NF‐κB, both important consequences of microbial signaling pathways, that drive cellular proliferation 2. Induction of SOCS3 in innate immune cells, such as IEC, is dependent upon MyD88, an adaptor protein used by all toll‐like receptors (TLR), except TLR3 7. In the absence of microbial signaling SOCS3 regulation of IEC turnover may be redundant; SOCS3 expression in the intestine of germ free (GF) animals is lower than that seen in conventional animals (Rigby, unpublished). MyD88‐deficient and GF, mice are more susceptible to colonic injury, but do not develop spontaneous or carcinogen induced colonic tumours 1, 8, 9, 10. These contrasting findings highlight that epithelial cells, and our immune system, must balance beneficial and detrimental effects of commensal microflora and support the notion that TLR signaling permits repair and restitution, yet must be tightly regulated to protect against excessive repair which can lead to cancer. Chronic overexpression of SOCS3, such as that seen in IBD, may hamper the turnover of rapidly dividing cells such as IEC, leading to destruction of the intestinal epithelial lining and inflammation 2, 11, 12, 13. Indeed SOCS3 predicts mucosal relapse in ulcerative colitis patients 14. Collectively these data support our central hypothesis that SOCS3, which impacts upon multiple signaling pathways, is a key mediator of mucosal homeostasis.

As well as maintaining epithelial barrier function, IEC homeostasis determines the susceptibility of the host to helminth infection 15. A Th2‐/IL‐13‐ driven transient increase in IEC turnover has been shown to facilitate pathogen expulsion in the mouse model of whipworm (*Trichuris muris)* infection. A failure to mount a short‐term increase in IEC turnover leads to IEC hyper‐proliferation, inflammation, and Th1‐associated chronic parasitic infection. In contrast, an increase in IEC turnover during early infection acts like an “epithelial escalator” aiding physical expulsion of the worm 16. Thus, the ability to respond to infection by increasing rates of IEC proliferation and turnover in the acute phase, appears to determine susceptibility to infection. Indoleamine 2,3‐dioxygenase (IDO), a key enzyme in tryptophan metabolism, is increased in mice chronically infected with *T. muris* 17. Resistance to infection can be achieved in susceptible animals through inhibition of IDO. IDO inhibition leads to increased migration of cells up the crypts and expulsion of worms without increasing crypt depth 18, suggesting that IDO limits the “epithelial escalator,” promoting worm persistance and survival, presumably due to its immunosuppressive properties 19.

We propose that SOCS3, as a natural endogenous regulator of IEC turnover, balances the magnitude and/or duration of inflammatory signaling maintaining IEC homeostasis and therefore modulates resistance to helminth infection. It is noteworthy that most studies have focused on the role of SOCS3 in immune cells. Evaluation of the role of SOCS3 in IEC is crucial since epithelial cells represent the primary cell type exposed to microbes in the intestinal lumen and maintain the barrier. We sought to determine whether epithelial SOCS3, known to limit epithelial proliferation in intestinal injury and tumour models, impacts on susceptibility to helminth infection through influencing IEC turnover.

## Results

**SOCS3 expression is increased in the intestine of mice susceptible to *T. muris* infection**

**Figure 1:**
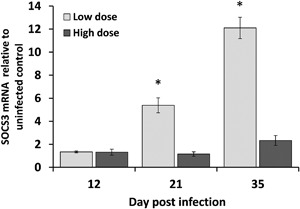
Mean SOCS3 mRNA in the cecal mucosa of mice infected with *T. muris*. SOCS3 was assessed at day 12, 21, and 35 post infection in C57BL/6 mice exposed to low or high dose infection. Expression was normalized to HMBS housekeeping gene, shown relative to uninfected controls ± SEM. *n* = 3 mice/group, *p < 0.0005.

The outcome of *T. muris* infection is dose‐dependent in C57BL/6 mice 20. High dose infection, with ≥40 eggs leads to expulsion within 35 days and a low‐dose (≤40 eggs) results in chronic infection. Following low dose infection SOCS3 mRNA (Fig. 1) was increased in the cecal epithelium, the primary location for egg hatching, and infection, compared to those infected with high dose. Differences in SOCS3 were not observed at 12 days post infection, however, increased SOCS3 expression was observed at day 21 while the explusion process is underway and persisted to day 35, when chronic infection is established. Therefore, increased SOCS3 may be mediating the inability to expel the parasite.

### IEC SOCS3 deficiency is associated with increased IEC turnover

**Figure 2:**
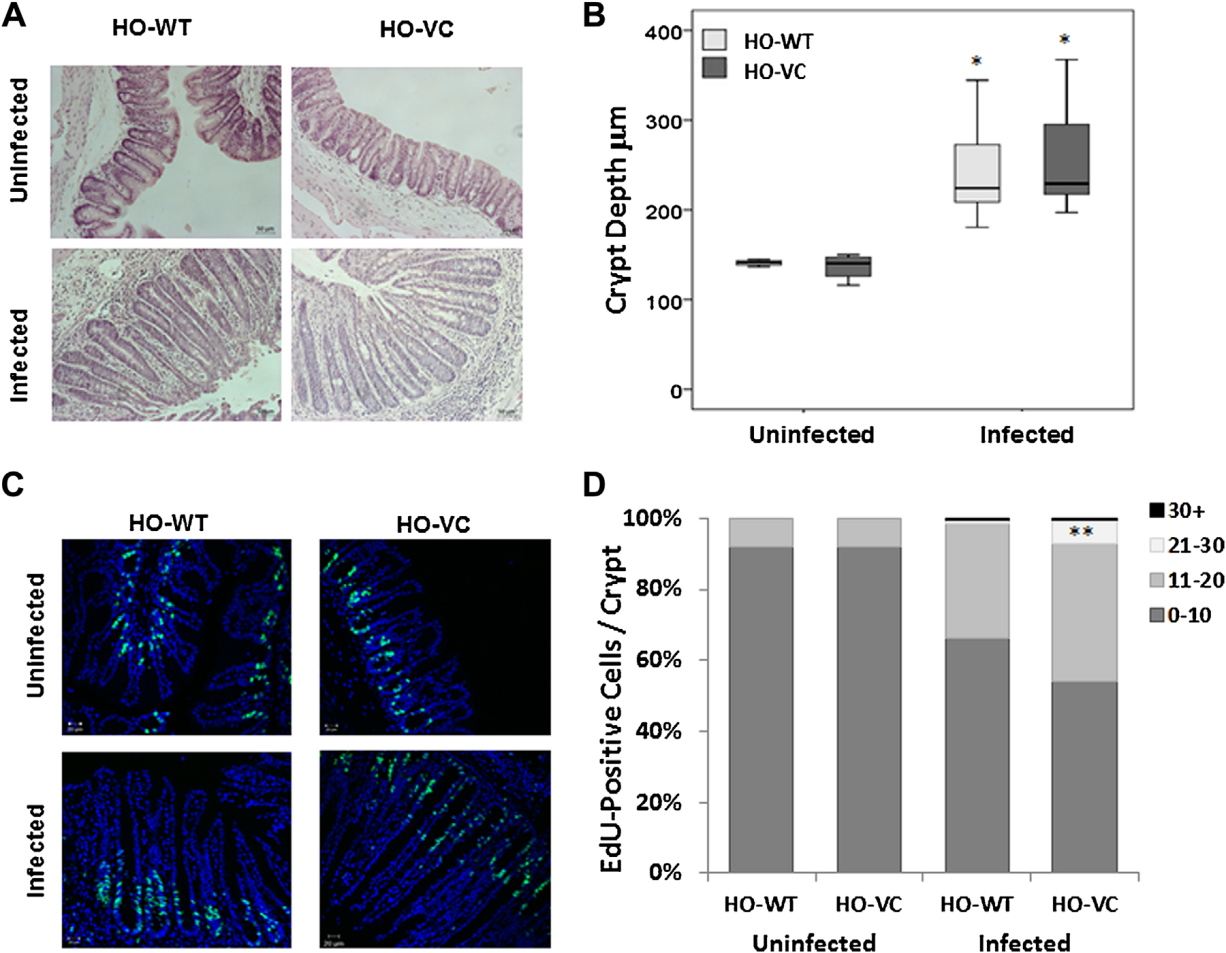
Epithelial cell turnover in knockout (HO‐VC) versus control (HO‐WT) mice. (A) Representive H&E stained sections of HO‐VC and HO‐WT cecum from uninfected and infected mice. (B) Box and Whisker plot showing average crypt depth (μm) of cecal tissue in uninfected and at 35 days post infection. Key: Light grey, HO‐WT; dark grey, HO‐VC. *p < 0.0001. (C) Representive EdU stained tissue showing proliferative cells (green) and counterstain (blue) of HO‐VC and HO‐WT cecum from uninfected and infected mice. (D) Percentage EdU positive cells in different regions of cecal crypts. Key indicates cell distance from bottom of crypt. **p = 0.008 Student's *t*‐test. Uninfected *n* = 3 HO‐WT and *n* = 5 HO‐VC. Infected *n* = 12 in both genotypes. Data acquired across two independent experiments.

Mice deficient in IEC SOCS3 (HO‐VC) and control homozygous littermates lacking the villin cre‐recombinase transgene (HO‐WT) were generated on the C57BL/6 background and infected with low dose *T. muris*. Confirming previous studies 15, low dose infection leads to chronic infection, and inflammation associated with increased crypt depth in cecum, regardless of genotype (Fig. 2A and B). Cell division occurs in the lower part of the crypt with new cells driving older cells further up the crypt until, under normal conditions, they reach the top and die. Therefore, in crypts undergoing greater numbers of cell divisions, EdU labeled cells appear further up the crypt. Upon infection EdU cells are consistently observed at higher positions within the crypt (Fig. 2D). Following infection a significantly higher percentage of EdU‐positive cells, were found in the region defined by cell number 21–30, in HO‐VC compared to HO‐WT mice (Fig. 2C and D, *p* = 0.008), supporting an increase in IEC turnover in SOCS3 deficient intestine. SOCS3 deficiency does not appear to impact upon crypt dynamics in uninfected mice.

### Worm burden is reduced in SOCS3 deficient mice

**Figure 3:**
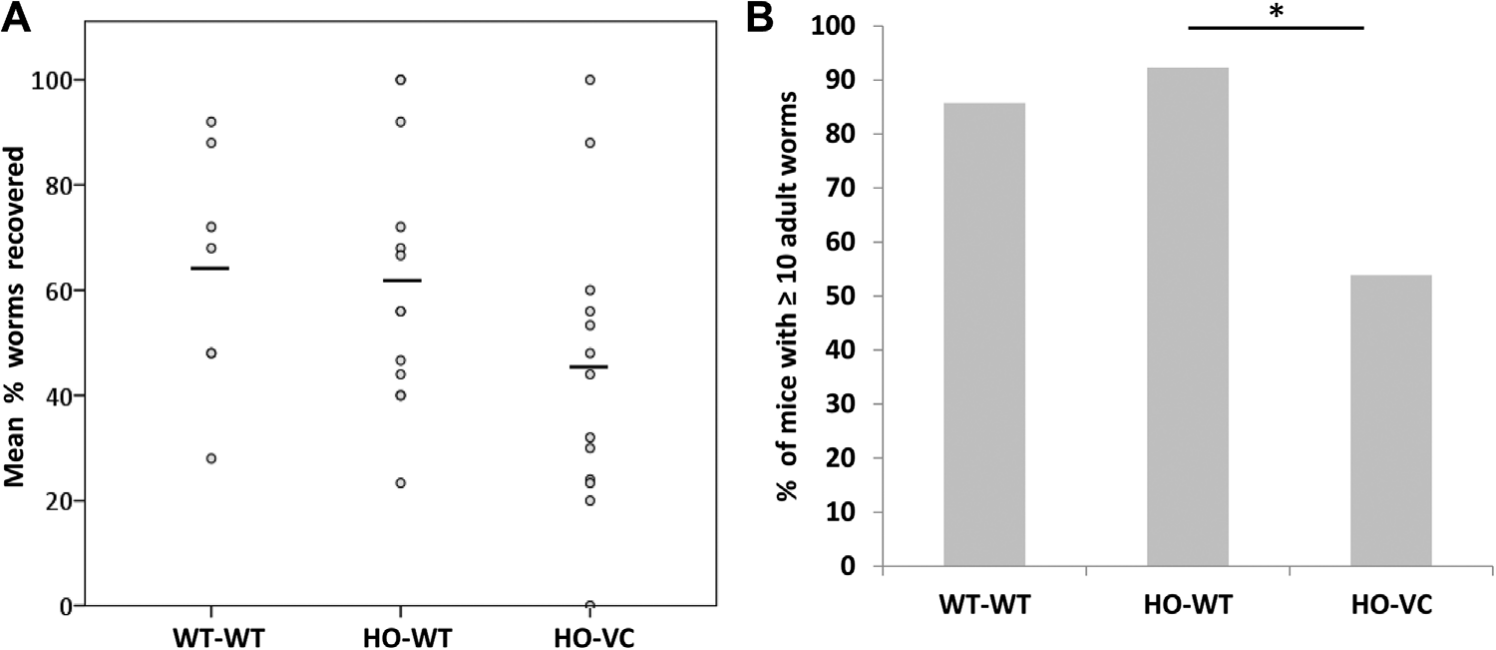
Worm burden in wild‐type (WT‐WT), control (HO‐WT), or knockout (HO‐VC) cecum. Mice were infected with 25–30 eggs and worm burdens assessed at 35 days post infection. (A) Individual worm burden expressed as the percentage of worms recovered of those that were inoculated (line denotes mean) and (B) percentage of mice with ≥10 adult worms. HO‐WT (*n* = 13), HO‐VC (*n* = 13), WT‐WT (*n* = 7), across two independent experiments. *p = 0.0365.

At 35 days post infection, *T. muris* worms are fully grown and established in the epithelial niche they occupy leading to chronic infection and inflammation. Comparison of mean worm burden in knockout (HO‐VC) mice to their control (HO‐WT) littermates supported a potential decrease in worm burden in HO‐VC (Fig. 3A), but numbers were variable. An accepted methodology to compare worm numbers in the context of variable worm burdens is to report the percentage of mice with low versus high numbers of worms 21. Thus, in order to assess differences in relative resistance the number of mice with low (<10 worms) versus high (≥10 worms) burdens were compared, revealing that a higher number of HO‐VC mice had lower worm burdens (Fig. 3B). This demonstrates a likely functional outcome of the increased IEC turnover in SOCS3 deficient intestine supporting that SOCS3 influences the rate of epithelial turnover. However, we were somewhat surprised to discover that IEC SOCS3 deficiency was not sufficient to eliminate all worms and confer resistance to *T. muris* infection.

**Increased mucosal IDO is associated with *T. muris* infection**

**Figure 4:**
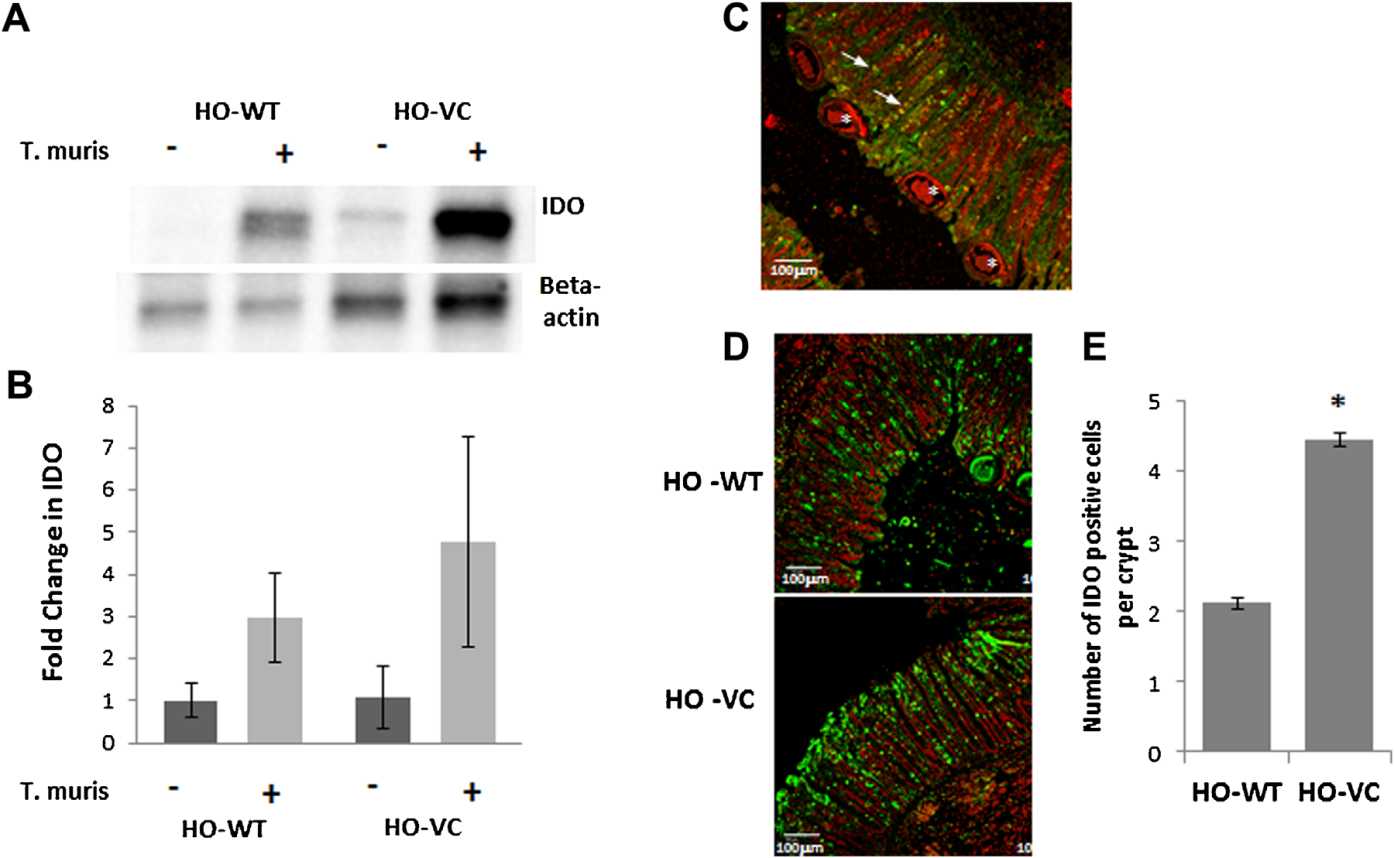
Mucosal IDO protein in HO‐VC and HO‐WT cecum from uninfected and infected mice at day 35 post inoculation. (A) Representative Western blot depicting mean IDO protein in cecal mucosal scrapes normalised to beta actin. (B) Mean IDO (±SEM) in cecal mucosa as determined by Western blot. Uninfected HO‐WT *n* = 6, HO‐VC *n* = 8. Infected HO‐WT *n *= 11, HO‐VC *n* = 7. (C) Representative sections of IDO (green) and muc2 (red) stained cecum of infected mice. Arrows point to dual‐labeled cells and asterix indicate worms. Magnification‐10×, scale bar = 100 μm. (D) Representative sections of IDO (green) staining in infected HO‐WT and HO‐VC cecal tissue, propidium iodide = red. Magnification‐ 10×, scale bar = 100 μm. (*n* = 5). (E) Quantification of IDO positive cells per crypt in infected cecum HO‐WT *n* = 8, HO‐VC *n* = 8, ±SEM, *p = 0.01.

Chronic *T. muris* infection is known to be associated with increased IDO and blocking IDO increases IEC turnover and aids expulsion 18. We therefore investigated the expression of IDO in cecal tissue using Western blotting (Fig. 4A and B) and immunofluorescence (Fig. 4C and D). IDO expression was increased following infection (Figure 4A and B, *p* = 0.05) and there was a trend for increased IDO following loss of SOCS3 in mucosa of uninfected mice (Fig. 4B), although this was not significant. The predominant producers of IDO in this model have previously been shown to be goblet cells 14. Indeed, immunofluorescence revealed that some, but not all, IDO was localized to goblet cells (Fig. 4C) and quantification of IDO positive cells per crypt, revealed an increased number of IDO‐positive cells in HO‐VC (4.5 ± 0.09) versus HO‐WT (2.1 ± 0.08) (Fig. 4D and E, *p* = 0.01). Chronic *T. muris* infection does lead to an increase in goblet cell number (due to crypt hyperplasia), but the proportion of goblet cells to total cells within a crypt does not alter (data not shown). Therefore, increased IDO following infection may be partly due to increases in goblet cell number, but differences between HO‐VC and HO‐WT is likely to be due to increased IDO production in SOCS3 deficient animals.

### IEC knockdown of SOCS3 is associated with increased IDO

**Figure 5:**
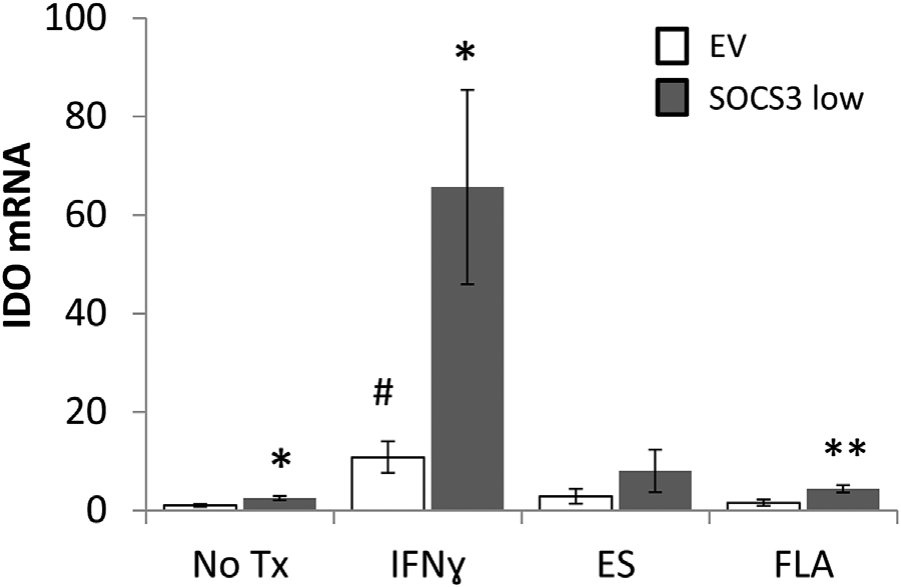
IDO mRNA in Human Intestinal Epithelial Cell (HIEC) lines. IDO mRNA in control empty vector (EV) and knockdown (SOCS3 low) epithelial cell lines ± SEM, following 2 h treatments. IDO shown relative to HMBS mRNA and EV no treatment (No Tx), *n* = 6 /group, across three independent experiments. EV versus SOCS3 low *p < 0.05, **p < 0.01; EV treatment versus No Tx, #*p* < 0.01.

To further investigate the impact of SOCS3 upon IDO expression, we utilized a human intestinal epithelial cell line (HIEC). Using shRNA, we achieved knockdown of SOCS3 both mRNA (60%) and protein (87%) in this cell line. We assessed IDO mRNA following treatment of cell cultures with IFN‐γ, purified *T. muris* excretory/secretory (ES) protein, or flagellin. In accordance with previous reports, IFN‐γ induced IDO expression 18, causing an 11 ± 3‐fold increase (*p* ≤ 0.01), but neither flagellin nor ES significantly upregulated IDO transcription above no treatment, in control (EV) cells (Fig. 5). SOCS3 depletion led to significant increase in IDO mRNA following all treatments, with the exception of ES which shows a trend. The most dramatic increase in IDO followed treatment with IFN‐γ, 66 ± 20‐fold increase relative to no treatment (*p* ≤ 0.0001), indicating that SOCS3 inhibits IDO transcription in IEC.

## Discussion

Dysregulation of epithelial homeostasis is apparent during insult to the intestine with a number of bacterial, viral, protozoan, and helminth infections 16, 22, 23. Such intestinal infections typically promote crypt cell hyperplasia and transient rapid IEC turnover is emerging as an important defence, likely serving to expel intestinal pathogens. If the increases in the kinetics of epithelial turnover are delayed or insufficient, chronic infection (and inflammation) may occur. Thus, understanding the mechanisms controlling IEC turnover, not only impact on our understanding of predisposition to inflammation and cancer, but also our resistance and/or susceptibility to intestinal infection.

Elevated SOCS3 expression is associated with susceptibility to chronic *T. muris* infection (Fig. 1). We therefore hypothesized, based on our previous evidence 2, 6, that SOCS3 was directly inhibiting the increased IEC turnover required to expel the parasites and thus mediating resistance or susceptibility in the C57BL/6 model. Indeed, loss of IEC SOCS3 led to increased IEC turnover and reduced worm burden, but the proliferative effects of loss of SOCS3, based on our previous work in an acute inflammation (DSS) model 2, were not as dramatic as expected; suggestive of a compensatory mechanism. We propose that the tempered increase in IEC turnover is due to increased IDO production in this model, attenuating the impact of immune‐cell produced inflammatory cytokines, which in turn increase IEC turnover, demonstrating the intricate interplay of immune signals regulating mucosal homeostasis.

Loss of SOCS3 enhanced IDO production in IEC. Evidence to support SOCS3 inhibition of IDO can be found from dendritic cells, in which SOCS3 has been shown to degrade IDO through proteosomal degradation 24. Loss of SOCS3 in DC results in the generation of tolerogenic DC and thus perpetuates immune evasion. In line with these findings, SOCS3 clearly inhibits both TLR and cytokine‐induced IDO production by IEC, as shown both in vitro and in vivo. The elevated IEC turnover is likely in reponse to cytokines produced by the adaptive (Th2/IL‐13‐mediated) immune response, as described in Bancroft et al. 25. Further support for the role of SOCS3 in mediating IEC responses to chronic inflammatory signals, can also be derived from our data showing no differences in IEC turnover at early time points, regardless of genotype.

Infection‐induced IDO protein is enhanced in SOCS3 deficient and sufficient‐mucosa. The lack of a distinct difference in IDO protein observed in the mucosal scrapes, between HO‐WT and HO‐VC is likely due to heterogeneity of the samples. For example, in addition to IEC and goblet cells, immune cells such as T‐cells, macrophages, and DC are also significant producers of IDO 26. IFN‐γ‐induced IDO was dramatically increased in SOCS3 knockdown IEC at the mRNA level. SOCS3 has previously been shown to mediate proteosomal degradation of IDO 27, however, it appears that in the absence of SOCS3‐mediated degradation, IEC transcribe more IDO. This may be because the products of IDO degradation are not fulfilling a negative feedback loop, or because several transcription factors are more active in the absence of SOCS3 28.

**Figure 6:**
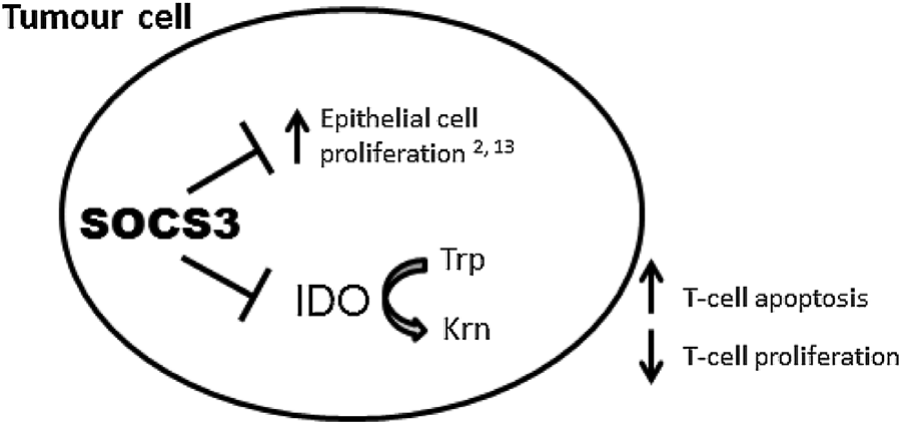
Proposed dual tumour‐suppressor role of SOCS3. Epithelial SOCS3 limits IEC proliferation 2, 6 and degrades IDO, limiting metabolism of tryptophan (Trp) to kynurenine (Krn), and therefore promoting immune surveillance by T‐cells.

The tumour suppressor activity of SOCS3 has previously been shown to act through “dampening” the impact of inflammatory cytokine signals, such as IL‐6, and suppressing proliferation 2, 29. Silencing of SOCS3, through promotor hypermethylation is a common feature of cancer cells 3, 4, however, it is not yet known how the resulting “highly proliferative” cells escape anti‐tumour immunity. The ability of IDO to limit T‐cell mediated immune surveillance promotes tumour development in multiple organs 30, 31. At tumour sites and tumour‐draining lymph nodes IFN‐γ, the most potent inducer of IDO 32, exerts anti‐proliferative effects on local T‐cells due to depletion of the essential amino acid tryptophan, and increases in tryptophan catabolites 26, 33. This leads to suppression of the host immune system and induces a state of tolerance toward the tumour. Because of this, IDO expression (in particular high expression) has been associated with poor prognosis and metastasis in a multitude of tumours 34, 35, 36. In addition to its ability to limit immune surveillance, IDO mediates its effects locally and independently of T‐cells. Both genetic and pharmacologic inhibition leads to colitis‐associated tumorigenesis in mice deficient in T‐cells 31. Therefore importantly, our findings suggest that SOCS3 not only limits pro‐tumourigenic proliferative signals 2, 13, but may also mediate its tumour suppressive effects through limiting IDO production and thus maintaining effective immune surveillance (Fig. 6).

In conclusion, the impact of SOCS3 upon intestinal epithelial homeostasis is context dependant as different models, ostensibly recapitulating acute, or chronic inflammatory states, act upon different biochemical pathways; demonstrating the intricate interplay of immune signals regulating mucosal homeostasis.

## Methods

### Animals

Following institutional ethical approval all experiments were conducted in accordance with University of Manchester Biological Services Facility regulations for animal husbandry and ethical guidelines and under Home Office licence in accordance with the Animals in Scientific Procedures Act (1986); PPL 40/3217 and 40/3633. Mice were housed in Specific Pathogen Free conditions, in individually ventilated cages with up to five littermates per cage, 12 h light/dark cycle and food, and water provided ad‐libitum. Mice homozygous for pLox‐SOCS3 modification (HO) derived on C57BL/6 background were mated with C57BL/6 mice hemizygous for Villin‐Cre (VC) transgene to generate mice with IEC specific SOCS3 knockout 2, 37. Validation of cell specific knockdown was previously shown in these animals 2. Male and female VC and WT litter mate mice homozygous for floxed SOCS3 (HO‐VC and HO‐WT respectively) were used as study animals. WT‐WT C57BL/6 animals, housed separately, were used as additional controls.

Genotyping used the following primers. WT or floxed SOCS3 alleles: 5′‐GTTTTCTCTGGGCGTCCTCCTA‐30 and 50‐TGGTACTCGCTTTTGGAGCTGAA‐30; VC transgene: 50‐GTGTGGGACAGAGAACAAACCG‐30 and 50‐TGCGAACCTCATCACTCGTTGC‐30; floxed or Cre‐excised SOCS3 alleles: 5′‐ACGTCTGTGATGCTTTGCTG‐30 and 50‐TCTTGTGTCTCTCCCCATCC‐30, yield a 740‐ or a 368‐bp fragment, respectively.

### *T. muris* infection and assessment

Six to eight week old HO‐VC, HO‐WT littermates or WT‐WT controls were infected with low dose (25–30) or high dose (∼200) *T. muris* eggs (Edinburgh isolate), in the morning, by oral gavage as previously described 38. Worm burdens (i.e., numbers) were assessed according to Else *et al.* 39 and worm excretory/secretory (ES) antigens were prepared as previously described 25.

### Histological and tissue samples

Intestinal tissue was collected for analysis at 0, 12, or 35 days post infection. Cecal tissue was fixed in 4% formaldehyde for 24 h prior to embedding in wax and sectioned for histological analysis including standard haematoxylin and eosin staining. Cecal mucosal scrapes were taken immediately after sacrifice, transferred to RNA or protein lysis buffer, and homogenized with syringe and needle before freezing.

### IEC proliferation and crypt depth

Edu (10 mg/kg) was administered by i.p. injection 90 min before sacrifice and EdU incorporating cells identified by fluoro‐ labeled azide using Click‐IT EdU imaging kit (Invitrogen, Life Technologies, Waltham, MA). The number and position of EdU‐labeled cells were evaluated in an average of 10 (at least 7) crypts per mouse in two independent experiments using Zeiss LSM Image Browser software. Crypt regions for assessment of cell position derived from Cliffe et al. 16 and Potten 40.

### Immunofluorescence

Heat‐Induced Epitope Retrieval using sodium citrate buffer (pH 6.0) was performed on dewaxed and rehydrated cecal sections. Sections were blocked in 10% normal serum in TBS + 1% BSA for 2 h at room temperature and incubated overnight at 4°C with primary antibody (rat IDO 1:400, Santa Cruz sc‐53978, Dallas, TX; rabbit SOCS3 1:1000, Abcam ab16030; Rabbit MUC2 1:200, Abcam ab76774). Secondary antibodies: AlexaFluor 488 goat anti‐rat IgG 1:500, AlexaFluor 488 donkey anti‐rabbit IgG 1:1000 or AlexaFluor 647 donkey anti‐rabbit IgG 1:1000 (Life Technologies) were incubated for 2 h and cells counterstained with propidium iodide or Hoeschst 33342, before mounting and viewing on a Zeiss LSM 510 confocal microscope. Quantification of IDO positive cells was carried out using ImageJ.

### Cell lines and culture

Normal (non‐transformed) human intestinal epithelial cells (HIEC), a gift from Jean‐Francois Beaulieu, Université de Sherbrooke, Canada, were maintained in Opti‐MEM reduced serum medium (Lonza) supplemented with 5% foetal bovine serum (Life Technologies), HEPES (1 mM, Sigma, St Louis, MO) and EGF (2 ng/ml, Life Technologies). Cells were grown in a humidified incubator at 37°C in 95% air: 5% CO_2_.

### Generation and confirmation of SOCS3‐knockdown cells

SOCS3 and non‐silencing shRNA GIPZ lentiviral constructs were purchased from Thermo Scientific (Waltham, MA) and were used to produce a SOCS3 knockdown and control HIEC cell lines. Each construct contained a puromycin resistance marker. HIEC cells were seeded at a concentration of 2 × 10^4^/well and were incubated with the respective lentivirus, according to manufacturer's instructions. Medium containing 1.35 μg/ml of puromycin was used to maintain transduced HIEC cells during culture and removed during experiments. Cells containing the SOCS3 (SOCS3^Low^) and non‐silencing constructs (SOCS3^Ev^ Ev‐Empty vector) were validated for SOCS3 mRNA (using qRTPCR) and protein. SOCS3^Low^ demonstrated a 60% ± 4 decrease in SOCS3 mRNA compared with SOCS3^Ev^ cells SOCS3 (95 bp) Forward: TCGATTCGGGACCAGCCCCC and Reverse: GAGCCAGCGTGGATCTGCG. SOCS3^Low^ cells showed an 87% ± 8 decrease in SOCS3 protein compared with SOCS3^Ev^ (rabbit primary antibody, Cell Signaling no. 2923).

### Assessment of IDO in cell cultures

SOCS3^Ev^ and SOCS3^Low^ HIEC cells were seeded at a concentration of 1 × 10^5^/ml, allowed to adhere for 24 h, then deprived of serum (and antibiotic) overnight prior to treatments. HIECs were treated with flagellin from *Salmonella typhimurium* (0.1 μg/ml, InvivoGen, San Diego, CA), interferon gamma (IFNɣ 10 ng/ml, Peprotech, Rocky Hill, NJ) or *Trichuris muris* excretory/secretory protein (ES, 0.1 mg/ml) for 2 h for mRNA and 4 h for protein analysis.

### qRT‐PCR

Reverse transcription (RT) was performed using the RevertAid Reverse Transcription kit (Thermo Scientific) with Anchored oligo (dT) (Thermo Scientific) and dNTPs to generate cDNA from 1 μg of RNA. Real‐time qPCR was performed using the SYBR® Green JumpStart™ Taq ReadyMix™ (Sigma), 0.5 μM forward and reverse primers (Sigma) on the CFX Real Time Thermocycler (Bio‐Rad, Hercules, CA). Cycling conditions were 95°C for 2 min, followed by 40 cycles of 95°C for 15 s, 67°C for 30 s and 72°C for 30 s for IDO; RPLPO and 35 cycles of 95°C for 20 s, 65°C for 30 s, and 72°C for 30 s for SOCS3 and HMBS. IDO, SOCS3, RPLPO, and HMBS primers were designed and checked for specificity by use of BLAST search from the National Center for Biotechnology Information (NCBI). The efficiency of all primers were in the acceptable range for use in the delta delta Ct method. Primer sequences were IDO (122 bp) Forward: CATGCTGCTCAGTTCCTCCA and Reverse: CCAGCATCACCTTTTGAAAGGA; RPLPO (142 bp) Forward: GCAATGTTGCCAGTGTCTG and Reverse: GCCTTGACCTTTTCAGCAA; SOCS3 (66 bp) Forward: GCACAAGCACAAAAATCCAGC and Reverse: AGAAGCCAATCTGCCCCTG and HMBS (70 bp) Forward: TGTGTTGCACGATCCTGAAAC and Reverse: CTCCTTCCAGGTGCCTCAGAA.

### qRT‐PCR analysis

Gene expression was calculated using the *R* *= *2^(−ΔΔCt)^ method. Changes in human IDO were normalized to those of the housekeeping gene, RPLP0. Fold‐change in IDO mRNA levels were calculated relative to SOCS3^Ev^ no treatment. Mouse SOCS3 values were normalized to housekeeping gene HMBS and levels were relative to uninfected (naïve) controls.

### Western blot

Cells or tissues were lysed in buffer (50 mM Tris, 150 mM NaCl, 0.5% sodium deoxycholate, 0.1% SDS, 1% IGEPAL) supplemented with phosphatase and protease inhibitors (Sigma). Cecal mucosal scrapes were passed through a blunt 18‐gauge needle (Becton Dickinson) and then centrifuged at 4°C at >10,000*g* for 10 min. Bradford reagent (Sigma) was used to determine protein concentrations. For both cells and mucosal scrapes: 30 μg of protein was boiled with 4x sample buffer (Bio‐Rad) and 10% 2‐Mercaptoethanol (Sigma) at 95°C for 5 min and SDS–PAGE performed. Proteins were transferred onto nitrocellulose membranes (Bio‐Rad), using the Trans‐Blot® Turbo™ Transfer System (Bio‐Rad). Membranes were blocked using 2% BSA in TBS + 0.1% Tween for 1 h at room temperature, then incubated overnight at 4°C in IDO primary antibody (Santa Cruz no. sc‐137012). Horseradish peroxidase‐conjugated goat anti‐mouse IgG secondary antibody (Santa Cruz no. sc‐2031) was used for visualizaton on the ChemiDoc™ MP Imaging System (Bio‐Rad). Densitometry analysis was carried out using Image Lab 5.2.1 software (Bio‐Rad) to determine IDO protein levels, normalized to alpha tubulin (Santa Cruz no. sc‐8035) or beta actin (sc‐130301) total protein.

### Statistical analysis

To determine statistical significance, *t*‐tests and ANOVA were performed using JMP software (version 12, SAS Institute), Tukey's post hoc test was used where appropriate. Fisher exact test was performed in SPSS. All analysis were performed on at least two independent experiments (with the exception of Fig. 1) and numbers are indicated in figure legends.

## Authors’ Contributions

RJR and KJE contributed in experimental design. EJS, EES, and JWD performed experiments. RJR, EJS, KJE, EES, and JWD contributed in analysis and interpretation of data. RJR, EJS, KJE, and EES contributed in drafting and revising manuscript. MDH generated and confirmed the SOCS3 knockdown and control cell lines by qPCR.

## Conflict of Interest

The authors confirm that there are no conflicts of interest.
